# Mitochondrial superclusters influence age of onset of Parkinson’s disease in a gender specific manner in the Cypriot population: A case-control study

**DOI:** 10.1371/journal.pone.0183444

**Published:** 2017-09-06

**Authors:** Andrea Georgiou, Christiana A. Demetriou, Alexandros Heraclides, Yiolanda P. Christou, Eleni Leonidou, Panayiotis Loukaides, Elena Yiasoumi, Dimitris Panagiotou, Panayiotis Manoli, Pippa Thomson, Maria A. Loizidou, Andreas Hadjisavvas, Eleni Zamba-Papanicolaou

**Affiliations:** 1 Neurology Clinic D, The Cyprus Institute of Neurology and Genetics, Nicosia, Cyprus; 2 The Cyprus School of Molecular Medicine, The Cyprus Institute of Neurology and Genetics, Nicosia, Cyprus; 3 Department of Primary Care and Population Health, University of Nicosia Medical School, Nicosia, Cyprus; 4 Neurology Clinic B, The Cyprus Institute of Neurology and Genetics, Nicosia, Cyprus; 5 Neurology Clinic C, The Cyprus Institute of Neurology and Genetics, Nicosia, Cyprus; 6 Limassol General Hospital, Limassol, Cyprus; 7 Private Neurology Clinic, Larnaca, Cyprus; 8 Department of Biological Sciences, University of Cyprus, Nicosia, Cyprus; 9 Department of Cardiovascular Genetics and the Laboratory of Forensic Genetics, The Cyprus Institute of Neurology and Genetics, Nicosia, Cyprus; 10 Centre for Genomic and Experimental Medicine, MRC Institute of Genetics and Molecular Medicine, University of Edinburgh, Edinburgh, United Kingdom; 11 Electron Microscopy and Molecular Pathology Department, The Cyprus Institute of Neurology and Genetics, Nicosia, Cyprus; Centre of Genomic & Post Genomics, ITALY

## Abstract

**Background:**

Despite evidence supporting an involvement of mitochondrial dysfunction in the pathogenesis of some neurodegenerative disorders, there are inconsistent findings concerning mitochondrial haplogroups and their association to neurodegenerative disorders, including idiopathic Parkinson’s disease (PD).

**Methods:**

To test this hypothesis for the Greek-Cypriot population, a cohort of 230 PD patients and 457 healthy matched controls were recruited. Mitochondrial haplogroup distributions for cases and controls were determined. Association tests were carried out between mitochondrial haplogroups and PD.

**Results:**

Mitochondrial haplogroup U was associated with a reduced PD risk in the Cypriot population. After pooling mitochondrial haplogroups together into haplogroup clusters and superclusters, association tests demonstrated a significantly protective effect of mitochondrial haplogroup cluster N (xR) and supercluster LMN for PD risk only in females. In addition, for female PD cases belonging to UKJT and R (xH, xUKJT) haplogroup, the odds of having a later age of onset of PD were 13 and 15 times respectively higher than the odds for female cases with an H haplogroup.

**Conclusion:**

Statistically significant associations regarding PD risk and PD age of onset were mostly detected for females thus suggesting that gender is a risk modifier between mitochondrial haplogroups and PD status / PD age of onset. The biological mechanisms behind this gender specificity remain to be determined.

## Introduction

Parkinson’s disease (PD) affects a substantial proportion of the elderly European population, including the Cypriot population. PD affects about 1% of the population over 60 years old in industrialized countries and this is expected to double in the next decades [1]. The prevalence and incidence rates of PD increase with age, having a steeper rise in individuals over 60 years old [2]. Sporadic PD accounts for at least 90% of the cases.

Moreover PD is a multifactorial disease caused by both genetic and environmental risk factors [3]. PD is characterized by selective loss of dopamine secreting neurons [1] and accumulation of Lewy bodies [4] in the brain and spinal cord. Midbrain dopaminergic neurons have a high rate of metabolic activity and are therefore dependent on the energy (ATP) released by oxidative phosphorylation in mitochondria. [5, 6] Consequently, mitochondrial dysfunction caused by genetic variants in both mitochondrial and nuclear DNA, can adversely affect neuronal function and lead to neurodegenerative diseases, such as Alzheimer’s disease (AD) and PD. Therefore PD pathogenesis has been strongly associated with both nuclear and mitochondrial genetic variants [7, 8].

Any population can be subdivided into phylogenetic clusters called mitochondrial haplogroups, which are characterized by specific mitochondrial polymorphisms that are inherited maternally through generations. The most common Western Eurasian mitochondrial haplogroups are H, I, J, K, T, U, V, W and X, which arose from the African super-haplogroup L3 [9]. Numerous studies have examined whether mitochondrial haplogroups are associated with the pathogenesis and risk of developing PD. Some mitochondrial haplogroups such as K and J have been found to be associated with a reduced risk of PD independently [10, 11] or after pooling them into the super-haplogroup UKJT in specific populations [12, 13]. However, there are some studies that did not find any association between mitochondrial haplogroups and PD. [14–16] Inconsistent results probably arise from the fact that participants in each study come from different populations with different mitochondrial genetic lineages and different frequencies of the aforementioned haplogroups. In addition, statistical variations in the sample size and approach used, such as choice of statistical tests, confounding factors, and corrections for multiple testing, may also explain a large proportion of the variation in the associations observed [16].

The Cypriot population results from the genetic admixture of several populations mainly from Eastern and Southern Europe and Western Asia (http://www.admixturemap.paintmychromosomes.com/) [17]. Therefore, due to high genetic admixture Cypriots have moderate genetic heterogeneity which is translated into a high diversity in mtDNA (mitochondrial DNA) gene pool. Interestingly, mitochondrial haplogroup K, which has been previously linked to lower PD risk, is particularly common in the Cypriot population, in frequencies much higher than other surrounding populations [18]. Herein we aim to investigate the presence of associations between mtDNA haplogroups and the risk for PD in the Cypriot population. A secondary aim, comprising a novel investigation, adding to the existing literature, is testing for any association between mitochondrial DNA haplogroups and PD age of onset and PD symptoms in the Cypriot population.

## Materials and methods

### Sample recruitment

A cohort of 230 PD patients was recruited from the Cyprus Institute of Neurology and Genetics (CING) and other medical centers across Cyprus (mean age 66.5 ± 10.5 years, mean age-of-onset 60.4 ± 11.4 years, 54.5% males and 45.5% females). Selection criterion for patient’s inclusion in the study was clinical diagnosis of PD by a board certified neurologist, followed by a clinical evaluation during the study, using the UPDRS rating scale by a board certified CING neurologist. Patients that had clinical signs suggestive of Parkinsonian syndromes were excluded. All patients were Greek-Cypriots.

Four hundred and fifty seven ethnically-matched controls were recruited using random cluster sampling from the general population. Cluster sampling included mailing letters of invitation to residences in randomly selected postal codes as well as visiting randomly selected medical/community centers across Cyprus. Individuals that did not suffer from any neurodegenerative disorder or cognitive impairment were invited to participate. Controls were ≥45 years old (mean age 65 ± 10.7 years, 50% males and 50% female).

The study was approved by the Cyprus National Bioethics Committee and all study participants, or their legal proxy, signed an informed consent form after detailed information regarding study participation was given.

Participants initially underwent an interview through an anonymized questionnaire that collected data regarding demographics, environmental exposures, medical history, lifestyle and other relevant characteristics such as diet, smoking, alcohol and coffee consumption. Subsequently, a peripheral blood sample or saliva was collected from all participants.

### Genotyping, determination and classification of mtDNA haplogroups

Total genomic DNA was isolated from peripheral-blood lymphocytes or saliva samples using the QIAamp DNA Blood Mini kit (Qiagen) following the manufacturer’s instructions. Polymerase chain reaction was used to amplify the entire mitochondrial DNA (mtDNA) Hypervariable Region 1 (HVR 1). Following PCR purification, direct sequencing was carried out using the BigDye Terminator cycle sequencing kit v 3.1 (Thermo Fisher Scientific). The produced sequence was aligned and compared against the revised Cambridge Reference Sequence (rCRS, Genbank: NC_012920) using SeqScape software (Thermo Fisher Scientific). The collective HVR 1 polymorphism information for all study participants was entered into an electronic database. Conversion of the annotated mitochondrial DNA HVR 1 variants into mitochondrial DNA haplogroups was performed using the 2.0 beta version of the web-based prediction tool HaploGrep (http://haplogrep.uibk.ac.at) [19] The predicted haplogroups were confirmed by testing the corresponding mtDNA coding region branch-defining SNPs by direct sequencing for all the samples (Table 1). The major mitochondrial haplogroups presented in Table 2 were further clustered into haplogroup-clusters based on common ancestors that they share and consequently common coding-region defining SNPs (Phylotree Build 17: www.phylotree.org) (Table 1) [20]. Haplogroup-clusters were then joined into superclusters based on previous studies, in order to compare our findings with other published studies that have used the same approach [12, 15]. As a result, the frequency of the independent variable was increased and accordingly the study power. The clusters created were L (xM, xN), M, N (including N, W, X and I) (xR), R (R*, R0), HV (HV, V), JT and U (including K) and the superclusters were LMN (including L, M, N, W, X and I), UKJT (including U, K, J and T) and R (including R*, R0, HV and V) (xH, xUKJT).

**Table 1:** MtDNA coding region branch-defining SNPs* for each haplogroup as used in the current study among a sample of Cypriot PD cases and controls.

	SNPs genotyped															
Haplogroup	1243 T/C	3594 C/T	3594 C/T	4580 G/A	6371 C/T	7028 T/C	10034 T/C	10238 T/C	10400 C/T	11467 A/G	10550 A/G	12612 A/G	12705 T/C	13368 G/A	14766 T/C	16126 T/C
**W**	**C**	C	C	G	C	T	T	T	C	A	A	A	T	G	T	T
**L3'4**	T	**C**	C	G	C	T	T	T	C	A	A	A	T	G	T	T
**L 0/1/2**	T	T	**T**	G	C	T	T	T	C	A	A	A	T	G	T	T
**V**	T	C	C	**A**	C	T	T	T	C	A	A	A	**C**	G	**C**	T
**X**	T	C	C	G	**T**	T	T	T	C	A	A	A	C	G	T	T
**H**	T	C	C	G	C	**C**	T	T	C	**G**	A	A	**C**	G	**C**	T
**I**	T	C	C	G	C	T	**C**	**C**	C	A	A	A	C	G	T	T
**N1**	T	C	C	G	C	T	T	**C**	C	A	A	A	C	G	T	T
**M**	T	C	C	G	C	T	T	T	**T**	A	A	A	T	G	T	T
**U**	T	C	C	G	C	T	T	T	C	**G**	A	A	**C**	G	T	T
**K**	T	C	C	G	C	T	T	T	C	**G**	**G**	A	**C**	G	T	T
**J**	T	C	C	G	C	T	T	T	C	A	A	**G**	**C**	G	T	**C**
R*	T	C	C	G	C	T	T	T	C	A	A	A	**C**	G	T	T
**T**	T	C	C	G	C	T	T	T	C	A	A	A	**C**	**A**	T	**C**
**HV**	T	C	C	G	C	T	T	T	C	A	A	A	**C**	G	**C**	T
**JT**	T	C	C	G	C	T	T	T	C	A	A	A	**C**	G	T	**C**

* Numbering according to GenBank number NC_012920

**Table 2:** Distribution of mtDNA haplogroups between Cypriot PD patients and controls and Odds Ratios (95% Confidence Intervals) showing their association with PD.

Haplogroup	Cases	Controls	Adjusted Model Association*	
	N (%)	N (%)	OR	p-value**
**H**	82 (35.7)	132 (28.9)	1	reference
**I**	3 (1.3)	12 (2.6)	0.47 (0.13–2.75)	0.62
**U**	21 (9.1)	70 (15.32)	0.49 (0.28–0.92)	0.02
**K**	22 (9.6)	52 (11.38)	0.74 (0.41–1.34)	0.32
**J**	17 (7.4)	26 (5.6)	1.22 (0.61–2.43)	0.56
**T**	27 (11.8)	51 (11.2)	0.93 (0.53–1.61)	0.79
**W**	5 (2.2)	15 (3.3)	0.61 (0.20–1.63)	0.36
**HV**	19 (8.3)	26 (5.7)	1.30 (0.61–2.47)	0.45
**X**	12 (5.2)	26 (5.7)	0.86 (0.40–1.86)	0.70
**N (xWXI, xR)**	5 (2.2)	16 (3.5)	0.55 (0.19–1.55)	0.26
**M**	4 (1.7)	9 (2.0)	0.91 (0.27–3.10)	0.88
**L (xM, xN)**	3 (1.3)	6 (1.3)	0.92 (0.22–3.84)	0.91
R (R*, **R0)**	10 (4.4)	16 (3.5)	1.12 (0.47–2.65)	0.80

*Logistic regression model adjusted for age, gender and maternal place of origin (PD:outcome, haplogroups: exposure)

** P-value nominal significance threshold = 0.05, Bonferroni adjusted significance threshold = 0.004

### Statistical analysis

Statistical analysis was performed using STATA V12 SE (StataCorp) statistical software package. Chi-square test with Yates correction was used to assess any significant difference in the overall mitochondrial haplogroup frequency distribution between PD cases and controls. The association between specific mitochondrial haplogroups and PD was further evaluated using binary logistic regression, in order to also adjust for potential confounders such as age, sex and place of origin. Haplogroups were tested individually and as superclusters that share common ancestors. The most common haplogroup (H) was used as the reference and this choice has the advantage that it is more homogeneous than a pooling of different haplogroups [21].

In the Logistic regression models, mitochondrial haplogroup variables were treated as the main exposure and PD status as the binary outcome. In addition, the same analysis was repeated with age of PD onset as the main outcome. Age of onset did not pass regression diagnostic tests for linear modelling assumptions, even after transformation attempts. Therefore the age of onset variable was separated into two categories, using the median age of onset (62 years) as a cut-off: earlier age of onset of PD (EOPD) with age of onset <62 years (reference category) and later age of onset of PD (LOPD) with age of onset ≥ 62 years (event category). Additionally logistic regression was used to evaluate the association between mitochondrial haplogroup frequencies and PD symptoms such as tremor and bradykinesia-dyskinesia.

Nominal statistical significance was established at p-value < 0.05 and Bonferroni correction was used to correct for multiple tests, dividing 0.05 by the number of mtDNA haplogroups, clusters or super-clusters that represent independent branches of the mtDNA phylogeny [16, 22].

## Results

Table 2 summarizes the distribution of mitochondrial haplogroups for both PD cases and controls. All nine major Western Eurasian haplogroups are present, as well as the more basal macro-haplogroups L, M, N and R. A major finding regarding haplogroup distribution in the Cypriot population is that mitochondrial haplogroup H is underrepresented whereas haplogroup K is overrepresented (Table 2), compared to other European countries, [10, 12, 14, 15].

Chi-square test did not reveal any significant differences in the overall haplogroup distribution between cases and controls (χ^2^ = 11.94, p-value = 0.45). Mitochondrial haplogroup U showed a nominally significant lower risk for PD (OR = 0.51, p-value = 0.03, 95%CI = 0.28–0.92). However, the association did not survive Bonferroni correction (Table 2).

The three superclusters and six clusters were tested for any association between cases and controls against haplogroup H, after adjusting for age, gender and maternal place of origin (Table 3). The association test for haplogroup clusters revealed a nominally significant protective association for U cluster and PD, when compared to haplogroup cluster HV. After stratification by gender U cluster did not preserve association in any of the two genders, while NWXI cluster displayed a significantly protective association for females, which survived Bonferroni correction (OR = 0.26, p-value = 0.006, 95% CI = 0.10–0.68). No significant difference between the supercluster distributions between cases and controls was observed. However, after stratification by gender, the supercluster LMN revealed a statistically significant protective effect for PD in comparison to the reference haplogroup H only for the female gender (OR = 0.34, p-value = 0.01, 95% CI = 0.15–0.77). This association was still statistically significant after correcting for multiple testing.

**Table 3:** Odds Ratios (95% Confidence Intervals) showing associations between mitochondrial haplogroup clusters and superclusters and PD.

	All participants		Males		Females	
**Haplogroup-clusters**	OR (95%CI)*	p-value**	**OR (95%CI)** ^∞^	p-value**	**OR (95%CI)** ^∞^	p-value**
**H**	1	reference	1	reference	1	reference
**HV (HV, V)**	1.29 (0.64–2.60)	0.48	2.02 (0.68–6.03)	0.21	0.94 (0.36–2.46)	0.90
**U (including K)**	0.61 (0.38–0.98)	0.03	0.68 (0.36–1.30)	0.24	0.58 (0.29–1.15)	0.12
**JT**	1.03 (0.64–1.65)	0.91	1.32 (0.68–2.57)	0.41	0.85 (0.43–1.68)	0.63
R (R*, **R0)**	1.01 (0.42–2.37)	0.98	0.77 (0.22–2.66)	0.68	1.78 (0.49–6.42)	0.38
**N (xR)**	0.65 (0.38–1.13)	0.10	1.23 (0.60–2.49)	0.57	0.26 (0.10–0.68)	0.006
**M**	0.92 (0.27–3.13)	0.90	0.76 (0.14–4.14)	0.75	1.08 (0.18–6.38)	0.94
**L (xM, xN)**	0.92 (0.27–3.13)	0.90	0.92 (0.08–10.74)	0.95	0.70 (0.12–4.18)	0.70
**Superclusters**	OR (95%CI)*	p-value***	**OR (95%CI)** ^∞^	p-value***	**OR (95%CI)** ^∞^	p-value***
**H**	1	reference	1	reference	1	reference
**UKJT**	0.78 (0.53–1.15)	0.21	0.91 (0.53–1.56)	0.73	0.71 (0.40–1.25)	0.23
**R (xH, xUKJT)**	1.19 (0.67–2.12)	0.55	1.11 (0.53–2.85)	0.80	1.32 (0.58–3.0)	0.58
**LMN**	0.69 (0.41–1.13)	0.14	1.11 (0.58–2.23)	0.75	0.34 (0.15–0.77)	0.01

*Logistic Regression Model for both genders adjusted for age, gender and maternal place of origin

^**∞**^ **Logistic Regression** Model for males and females separately adjusted for age and maternal place of origin

** P-value nominal significance threshold = 0.05, Bonferroni adjusted significance threshold = 0.007

*** P-value nominal significance threshold = 0.05, Bonferroni adjusted significance threshold = 0.017

Age of onset was converted into a binary variable consisting of 103 EOPD and 118 LOPD cases. Superclusters were then tested for any association with the age of onset. Patients that belonged to the supercluster UKJT had a significant association with LOPD compared to the reference haplogroup H (OR = 4.09, p-value = 0.005, 95% CI = 1.5–10.9) and after stratification by gender, this association appeared even stronger in females (Table 4). More specifically female cases belonging to UKJT mitochondrial supercluster had 13 times higher probability of having LOPD compared to those with an H haplogroup and the association survived Bonferroni correction. Additionally there was a statistically significant association between the R supercluster and a later age of onset only for female patients (see Table 4). These results suggest that gender is an effect modifier for PD and this was further supported for UKJT when an interaction term was entered into the model with age of onset as the outcome, resulting in a statistically significant interaction (p_interaction_ for UKJT = 0.02). For R (xH, xUKJT) the interaction term approached but did not reach statistical significance (p_interaction_ for R (xH, xUKJT) = 0.056).

**Table 4:** Odds Ratios (95% Confidence Intervals) showing associations between Cypriot mitochondrial haplogroup superclusters and age of PD onset (EOPD versus LOPD).

	All participants		Males		Females	
mtDNA Superclusters	OR (95%CI)*	p-value**	OR (95%CI)*	p-value**	OR (95%CI)*	p-value**
**H**	1	reference	1	reference	1	reference
**UKJT**	4.07 (1.5–10.9)	0.005	1.42 (0.3–6.1)	0.64	13.55 (2.8–64.7)	0.001
**LMN**	0.69 (0.2–2.6)	0.58	0.29 (0.05–1.8)	0.19	2.49 (0.2–25.1)	0.44
**R (xH, xUKJT)**	3.42 (0.96–12.2)	0.06	1.14 (0.2–6.6)	0.88	15.84 (1.7–144)	0.014

* Logistic Regression Model adjusted for age and maternal place of origin

** P-value Nominal significance threshold = 0.05, Bonferroni adjusted significance threshold = 0.017

PD symptoms such as tremor and bradykinesia did not show any statistically significant association with any mitochondrial haplogroup or supercluster (S1 and S2 Tables).

## Discussion

The hypothesis whether mitochondrial DNA polymorphisms are associated with PD pathogenesis is controversial. In this study we found an association between the mitochondrial haplogroup U (xK) and PD, with individuals belonging to the specific haplogroup having ~50% lower likelihood of having PD in our sample. None of the previous studies have reported a statistically significant association between the U haplogroup and PD. Additionally, this association did not survive Bonferroni correction, and thus the possibility of it being a false positive result cannot be eliminated.

However, there are numerous other studies that have found evidence for association between PD and haplogroups U, J and K. Van der Walt et al [2003] have reported a lower frequency of haplogroups J and K in PD cases compared to controls [21]. A larger study 2 years later by Ghezzi *et al*. [2005] has found that haplogroup K, but not J was significantly underrepresented in cases than controls, especially in males [10]. Furthermore, Gaweda *et al*. [2008] have reported a protective association between haplogroup J and PD only in males [11]. Yet, this finding is inconsistent with two other studies, concerning other European populations that did not detect any association between mitochondrial haplogroups and PD [14–16].

Inconsistent results in the reported studies probably arise from the fact that participants in each study come from different populations with different genetic lineages and different population frequencies of the aforementioned haplogroups, as well as their subclades. For example, subclades of mitochondrial Haplogroup U among Cypriots are not the same as U subclades found in continental Europe [18, 23, 24].

In addition, the inconsistent and contradictory results on associations between mitochondrial haplogroups and PD may also be explained by the fact that most of the available studies have a small sample size, lending themselves to both false positive and false negative findings (i.e. type I and II errors). Furthermore, statistical variations in the approach used, such as confounding factors included, and corrections for multiple testing, may also explain a large proportion of the variation in the associations observed [16].

Even though some of the aforementioned studies did not adjust for any confounders, the majority of studies has adjusted their analyses, for age and gender, but has not performed population stratification. Adjusting for confounders is very important in terms of false positive results as demonstrated in the present study where adjustment for confounders explained away mitochondrial cluster N (xR) and supercluster LMN’s significant protective association with PD (S3 Table).

A meta-analysis study aimed to resolve at least part of the inconsistency of studies by combining the data from multiple association studies concerning mitochondrial haplogroups and a variety of diseases such as PD, AD, breast cancer and Type 2 Diabetes. The results of this study showed that individuals with the haplogroup K had a significantly decreased risk for PD onset. Also haplogroups J and T are associated with protection against PD, while haplogroup H is associated with an increased susceptibility to age-related diseases. Haplogroups H, K and J are associated with multiple diseases, which suggest that the genetic variations that define those haplogroups could have a pleiotropic impact on human diseases. The exact biological mechanisms involved in this pleiotropic impact of mtDNA variants remains unclear. Further functional studies on mitochondrial pathways such as oxidative phosphorylation and apoptosis could shed light on this [25].

In order to increase the frequency of the independent variable and consequently increase the study power, mitochondrial haplogroups were combined into clusters and super-clusters of haplogroups that share common ancestors. Combination of haplogroups into haplogroup clusters and super-clusters revealed once more an association only for cluster U (including K) and PD. After stratification by gender, NWXI haplogroup cluster and LMN super-cluster showed evidence for association with PD only for females, both of which survived Bonferroni correction. This suggests that NWXI mitochondrial cluster drives the association between LMN supercluster and PD among the female cases.

Finally, UKJT super-cluster showed a statistically significant association with a later age of onset of PD when compared to the common haplogroup H. This association of the UKJT supercluster and PD was even stronger in female cases and the association survived multiple testing correction. Female PD cases who belong to cluster UKJT have 13.5-fold higher likelihood of being diagnosed at a later age (>62 years) compared to those belonging to haplogroup H. Additionally R (xH, xUKJT) haplogroup showed evidence for a protective association in respect to the age of onset of PD, only among females. This suggests that gender is an effect modifier between mitochondrial haplogroups and the age of onset of PD and could explain at least in part, the fact that females have on average a later age of onset of PD than males [26]. Therefore there might be specific mitochondrial polymorphisms which are shared by certain mtDNA haplogroups that affect mitochondrial function to a much greater extent in females than in males. This possibility might be explained by the fact that estrogen receptors (ERs), activated by estrogen female hormones, bind to mitochondrial estrogen responsive elements (mtEREs) and this binding of ERs to the mtEREs increases the expression of mitochondrial genes that are associated with the electron transport chain [27–29]. Certainly this is only a hypothesis, which should be investigated further. However, there are a number of other publications reporting female genetic distribution bias and maternal inheritance bias on mtDNA in PD patients. For example, in Chu et al. (2015), a susceptibility locus combined with haplotype has been shown to influence PD risk, only in females [30].

This study is the first investigation of mtDNA haplogroups and PD risk in the Cypriot population and, to the best of our knowledge, the first investigation of the association between mtDNA haplogroups and PD age of onset and PD symptoms. This is a nationwide investigation, recruiting cases and controls from all districts of Cyprus, minimizing thus the risk of bias due to founder effect and maximizing the findings’ external validity. Furthermore, the mtDNA haplogroup designations were not based merely on predictions from publicly available software, but they were confirmed through further SNP analyses. Lastly, the study serves to highlight the most important challenges regarding existing studies in the field of mtDNA haplogroups and PD risk, especially the importance of correcting for confounders and multiple testing, as well as gender stratification.

The current study is not without limitations, its small sample size leading to low study power being perhaps its greatest restriction. This is particularly relevant to the age of onset regression analysis. A larger sample size would be more preferable, in order to detect even small associations between certain haplogroups and PD, if they existed [31]. However, given the fact that Cyprus is a small country and the fact that PD is not a common disease, although it affects a substantial proportion of the elderly population, a larger sample size comprising of more cases, was almost impossible to recruit. The bradykinesia and dyskinesia of PD patients also contributed to the limited sample size since it often translated into a reduced willingness to participate in the study.

In conclusion, in this novel investigation, mitochondrial haplogroup U was associated with a reduced PD likelihood in the Cypriot population. Furthermore, there was a significant protective association between haplogroup cluster NWXI and PD only in females. Most importantly, a statistically significant protective effect of supercluster UKJT and R (xH, xUKJT) with respect to a later age of onset of PD when compared with haplogroup H was demonstrated for the first time, in female PD patients. The biological mechanisms behind this novel finding remain to be determined in future investigations.

## Supporting information

S1 Table Odds Ratios (95% Confidence Intervals) showing associations between Cypriot mitochondrial superclusters and PD symptoms. (DOCX)

S2 Table Odds Ratios (95% Confidence Intervals) showing associations between Cypriot mitochondrial superclusters and PD symptoms, after stratification by gender. (DOCX)

S3 Table Odds Ratios (95% Confidence Intervals) showing associations between Cypriot mitochondrial clusters and superclusters and PD, without adjusting for any confounders. (DOCX)

S4 Table Odds Ratios (95% Confidence Intervals) showing associations between MtDNA coding region branch-defining SNPs and PD, after adjusting for age, gender and maternal place of origin. (DOCX)
