# Supplementary material for: Mitochondrial superclusters influence age of onset of Parkinson’s disease in a gender specific manner in the Cypriot population: A case-control study
Source: PLoS One. 2017 Sep 6;12(9):e0183444. doi: 10.1371/journal.pone.0183444 (PMC5587277; doi:10.1371/journal.pone.0183444)
Supplement: S1 Table — (DOCX) [file pone.0183444.s001.docx]

**Table S1** Odds Ratios (95% Confidence Intervals) showing associations between Cypriot mitochondrial superclusters and PD symptoms

|  | **Tremor** | | | **Rigidity-Bradykinesia** | |
| --- | --- | --- | --- | --- | --- |
| **Haplogroup** | | **OR (95%CI)*** | **p-value**** | **OR (95%CI)*** | **p-value**** |
| **H** | | 1 | reference | 1 | reference |
| **UKJT** | | 0.89 (0.33-2.44) | 0.83 | 1.10 (0.36-3.33) | 0.87 |
| **LMN** | | 0.70 (0.21-2.29) | 0.56 | 0.89 (0.25-3.22) | 0.86 |
| **R** | | 1.66 (0.32-8.68) | 0.55 | 0.64 (0.16-2.56) | 0.52 |

*Model adjusted for age, gender and maternal place of origin

** Nominal significance threshold=0.05, Bonferroni adjusted significance threshold=0.017
