# Supplementary material for: Mitochondrial superclusters influence age of onset of Parkinson’s disease in a gender specific manner in the Cypriot population: A case-control study
Source: PLoS One. 2017 Sep 6;12(9):e0183444. doi: 10.1371/journal.pone.0183444 (PMC5587277; doi:10.1371/journal.pone.0183444)
Supplement: S2 Table — (DOCX) [file pone.0183444.s002.docx]

|  | **Tremor** | | | | **Rigidity-Bradykinesia** | | | |
| --- | --- | --- | --- | --- | --- | --- | --- | --- |
|  | **Males** | | **Females** | | **Males** | **Females** |  |  |
| **Haplogroup** | **OR (95%CI)*** | **p-value**** | **OR (95%CI)*** | **p-value**** | **OR (95%CI)*** | **p-value**** | **OR (95%CI)*** | **p-value**** |
| **H** | 1 | reference | 1 | reference | 1 | reference | 1 | reference |
| **UKJT** | 1.12 (0.3-4.2) | 0.86 | 0.90 (0.2-4.8) | 0.90 | 1.25 (0.2-7.1) | 0.80 | 0.60 (0.1-2.8) | 0.52 |
| **LMN** | 0.98 (0.2-4.5) | 0.98 | 0.80 (0.1-6.6) | 0.84 | 1.02 (0.1-7.5) | 0.98 | 0.22 (0.0-1.6) | 0.13 |
| **R** | 2.77 (0.3-27.4) | 0.38 | 1.16 (0.1-15.1) | 0.91 | 0.48 (0.1-3.7) | 0.48 | 0.45 (0.1-4.1) | 0.48 |

**Table S2** Odds Ratios (95% Confidence Intervals) showing associations between Cypriot mitochondrial superclusters and PD symptoms, after stratification by gender

*Model adjusted for age and maternal place of origin

** Nominal significance threshold=0.05, Bonferroni adjusted significance threshold=0.017
