# Supplementary material for: Mitochondrial superclusters influence age of onset of Parkinson’s disease in a gender specific manner in the Cypriot population: A case-control study
Source: PLoS One. 2017 Sep 6;12(9):e0183444. doi: 10.1371/journal.pone.0183444 (PMC5587277; doi:10.1371/journal.pone.0183444)
Supplement: S3 Table — (DOCX) [file pone.0183444.s003.docx]

|  | **All participants (Baseline model)** | | |
| --- | --- | --- | --- |
| **Haplogroup** | **OR (95%CI)** | **p-value*** | |
| **H** | 1 | reference | |
| **UKJT** | 0.71 (0.49-1.04) | 0.08 | |
| **R (R*, R0)** | 1.08 (0.62-1.87) | 0.78 | |
| **LMN** | 0.61 (0.37-0.99) | 0.05 | |
| **Haplogroup** | **OR (95%CI)** | | **p-value**** |
| **H** | 1 | | reference |
| **HV (HV, H, V)** | 1.19 (0.61-2.31) | 0.62 | |
| **L** | 0.82 (0.19-3.27) | 0.76 | |
| **M** | 0.71 (0.21-2.37) | 0.58 | |
| **NWXIN (xR)** | 0.58 (0.34-0.98) | 0.04 | |
| **R (R*, R0)** | 1.07 (0.46-2.46) | 0.88 | |
| **JT** | 0.91 (0.58-1.42) | 0.69 | |
| **U (including K)** | 0.57 (0.37-0.87) | 0.01 | |

**Table S3** Odds Ratios (95% Confidence Intervals) showing associations between Cypriot mitochondrial clusters and superclusters and PD, without adjusting for any confounders

*Nominal significance threshold=0.05, Bonferroni adjusted significance threshold=0.017

**Nominal significance threshold=0.05, Bonferroni adjusted significance threshold=0.007
