# Supplementary material for: Mitochondrial superclusters influence age of onset of Parkinson’s disease in a gender specific manner in the Cypriot population: A case-control study
Source: PLoS One. 2017 Sep 6;12(9):e0183444. doi: 10.1371/journal.pone.0183444 (PMC5587277; doi:10.1371/journal.pone.0183444)
Supplement: S4 Table — (DOCX) [file pone.0183444.s004.docx]

| MtDNA coding region branch-defining SNP | OR (95% CI)^*^ | p-value^**^ |
| --- | --- | --- |
| **1243 T/C** | 0.64 (0.23-1.80) | 0.40 |
| **3594 C/T** | 2.11 (0.23-19.60) | 0.51 |
| **4580 G/A** | 6.55 (1.23-34.80) | 0.03 |
| **6371 C/T** | 0.93 (0.45-1.93) | 0.84 |
| **7028 T/C** | 1.25 (0.88-1.78) | 0.21 |
| **10034 T/C** | 0.54 (0.15-1.95) | 0.35 |
| **10238 T/C** | 0.60 (0.27-1.35) | 0.22 |
| **10400 C/T** | 1.06 (0.32-3.53) | 0.92 |
| **11467 A/G** | 0.63 (0.42-0.96) | 0.03 |
| **10550 A/G** | 0.86 (0.50-1.49) | 0.60 |
| **12612 A/G** | 1.40 (0.73-2.67) | 0.31 |
| **12705 T/C** | 1.20 (0.60-2.41) | 0.61 |
| **13368 G/A** | 1.10 (0.66-1.83) | 0.71 |
| **14766 T/C** | 1.38 (0.99-1.94) | 0.06 |
| **16126 T/C** | 1.23 (0.81-1.87) | 0.34 |

*Logistic regression model adjusted for age, gender and maternal place of origin (PD: outcome, MtDNA coding region branch-defining SNP: exposure)

** P-value nominal significance threshold=0.05
